# *Enterococcus faecalis rnc* gene modulates its susceptibility to disinfection agents: a novel approach against biofilm

**DOI:** 10.1186/s12903-022-02462-1

**Published:** 2022-09-20

**Authors:** Mengying Xia, Niya Zhuo, Shirui Ren, Hongyu Zhang, Yingming Yang, Lei Lei, Tao Hu

**Affiliations:** Department of Preventive Dentistry, West China Hospital of Stomatology, Key Laboratory of Oral Diseases, Sichuan University, NO. 14 Third Section Renmin South Road, Chengdu, China

**Keywords:** *Enterococcus faecalis*, Biofilm, *rnc*, Extracellular polysaccharide, Traditional Chinese medicine

## Abstract

**Background:**

*Enterococcus faecalis* (*E. faecalis*) plays an important role in the failure of root canal treatment and refractory periapical periodontitis. As an important virulence factor of *E. faecalis*, extracellular polysaccharide (EPS) serves as a matrix to wrap bacteria and form biofilms. The homologous *rnc* gene, encoding Ribonuclease III, has been reported as a regulator of EPS synthesis. In order to develop novel anti-biofilm targets, we investigated the effects of the *rnc* gene on the biological characteristics of *E. faecalis*, and compared the biofilm tolerance towards the typical root canal irrigation agents and traditional Chinese medicine fluid Pudilan.

**Methods:**

*E. faecalis rnc* gene overexpression (*rnc*+) and low-expression (*rnc*−) strains were constructed. The growth curves of *E. faecalis* ATCC29212, *rnc*+, and *rnc*− strains were obtained to study the regulatory effect of the *rnc* gene on *E. faecalis*. Scanning electron microscopy (SEM), confocal laser scanning microscopy (CLSM), and crystal violet staining assays were performed to evaluate the morphology and composition of *E. faecalis* biofilms. Furthermore, the wild-type and mutant biofilms were treated with 5% sodium hypochlorite (NaOCl), 2% chlorhexidine (CHX), and Pudilan. The residual viabilities of *E. faecalis* biofilms were evaluated using crystal violet staining and colony counting assays.

**Results:**

The results demonstrated that the *rnc* gene could promote bacterial growth and EPS synthesis, causing the EPS-barren biofilm morphology and low EPS/bacteria ratio. Both the *rnc*+ and *rnc*− biofilms showed increased susceptibility to the root canal irrigation agents. The 5% NaOCl group showed the highest biofilm removing effect followed by Pudilan and 2% CHX. The colony counting results showed almost complete removal of bacteria in the 5% NaOCl, 2% CHX, and Chinese medicine agents’ groups.

**Conclusions:**

This study concluded that the *rnc* gene could positively regulate bacterial proliferation, EPS synthesis, and biofilm formation in *E. faecalis*. The *rnc* mutation caused an increase in the disinfectant sensitivity of biofilm, indicating a potential anti-biofilm target. In addition, Pudilan exhibited an excellent ability to remove *E. faecalis* biofilm.

**Supplementary Information:**

The online version contains supplementary material available at 10.1186/s12903-022-02462-1.

## Introduction

Periapical periodontitis is an inflammatory disease, which occurs in the periapical tissues and is caused by microbial infection in dental pulp [1, 2]. The persistent infection of the apical root canal system is a risk factor for the clinical and radiographical signs of periapical periodontitis [3]. Gram-positive bacteria have been found in about 85% of the teeth treated with root canal therapy; among them, *Enterococcus faecalis* (*E. faecalis*) was detected in persistent endodontic infections, ranging from 24 to 77% [4, 5]. Recently, *E. faecalis* has been paid more attention due to its dominant role in the formation of extra radicular biofilm and periapical lesions [6]. According to the study by Barbosa-Ribeiro et al., *E. faecalis* was the most abundant bacteria in the teeth with endodontic treatment failure and was also associated with the periapical lesions of over 3-mm size [7]. It colonizes the biofilms, invades the dentinal tubules, and resists nutritional deprivation, thereby causing therapeutic failure and heavy economic burdens [4, 8].

*E. faecalis* is a Gram-positive coccus, which is homologous with the dental caries pathogen *Streptococcus mutans* (*S. mutans*). The formation of biofilms results in the adhesion and aggregation of bacteria cells as well as increased resistance to root canal irrigants. The VicRK two-component signal transduction system is a key regulator in the synthesis of exopolysaccharide (EPS) in *S. mutans*. A previous study reported that the *rnc* gene, encoding ribonuclease III (RNase III), could promote the EPS synthesis and alter the morphology of biofilm [9]. However, the *rnc* gene function has rarely been detected in *E. faecalis*. Our previous study showed that *rnc* could repress *vicRKX* expressions at the post-transcriptional level via microRNA-size small RNAs (msRNAs) [10]. The WalRK signal transduction system in *E. faecalis*, which is homologous to VicRK, could also regulate EPS synthesis. It was reported that inhibiting the biofilm formation-related gene *walR* could reduce EPS synthesis and enhance the susceptibility of *E. faecalis* biofilms to chlorhexidine (CHX) [11]. Therefore, regulating the metabolism of biofilms might be a feasible way for eliminating *E. faecalis* biofilm infections. Due to the homology of the *rnc* gene in *E. faecalis* with that in *S. mutans*, it was speculated that the *rnc* gene could regulate the morphology of biofilms by promoting the EPS synthesis in *E. faecalis*.

Sodium hypochlorite (NaOCl) has been widely used in the irrigation of root canal due to its excellent antibacterial properties and ability to remove organic components and tissue remnants [12]. However, it has also raised concerns due to its cytotoxic effects on the periapical and pulp tissues [13]. CHX has also been widely used for the irrigation of root canal due to its excellent antibacterial activity. However, it is unable to dissolve the tissue remnants, which restricts its applications as a standard irrigation agent [14]. The current irrigation agents cannot be considered an ideal choice individually. Therefore, exploring new irrigation agents is needed.

Traditional Chinese medicine (TCM) has a history of thousands of years. These natural medicines are increasingly applied for the treatment of oral diseases. Pudilan is a TCM fluid, which has anti-inflammatory and antibacterial effects. It is made up of the extracts of multiple cold and calm herbs, including Scutellaria baicalensis root, Taraxacum mongolicum, Bunge corydalis herb, and Isatis indigotica [15]. Its anti-inflammatory effects have been confirmed in several classic inflammatory models [16]. It has also been applied to cure oral diseases, such as mild recurrent aphthous ulcers and chronic gingivitis [17, 18]. The active ingredient in Pudilan has proved to inhibit the production of varies inflammatory factors, such as periodontitis target IL-1β [19, 20]. Nevertheless, the antibacterial effects of Pudilan on *E. faecalis* or periapical periodontitis have not been investigated yet. Therefore, this study was aimed to explore the potential targets for the disinfection of *E. faecalis* biofilms and also explore the clinical alternative drugs. The main objectives of this study were as follows: (1) to construct and verify the *rnc* overexpression and low-expression mutant strains of *E. faecalis*; (2) to detect the regulatory effect of *rnc* on the morphology of biofilm and EPS production; and (3) to evaluate the *rnc* modulated susceptibility of *E. faecalis* biofilms to root canal irrigation agents and Pudilan.

## Materials and methods

### Strains and culture conditions

*Enterococcus faecalis* ATCC 29212 strain was provided by the State Key Laboratory of Oral Diseases (China) and stored at − 80 °C. The *rnc* gene sequence was acquired from NCBI (Gene ID: 60892348). The *rnc* overexpression recombinant plasmid was designed and synthesized by adding promoters upstream of the *rnc* gene and cloning them into a spectinomycin-resistant shuttle vector pDL278. The recombinant plasmids were transformed into the *E. faecalis* ATCC 29212 strain through the chemical transformation method using 1 μg/mL competence-stimulating peptides (CSP) and the *rnc* overexpression mutant strain (*rnc*+) was established [10]. In order to establish the *rnc* low-expression mutant strains (*rnc*−), the reverse complementary sequences of *rnc* were designed and introduced into the pDL278 vector with promoter sequences [21–23]. Then, the plasmids were transformed into *E. faecalis* ATCC 29212 strain similar to that of *rnc*+ strains. The strains were cultured in brain heart infusion (BHI) broth (Difco, Detroit. MI. USA) at 37 °C under anaerobic conditions (80% N_2_, 10% H_2_, 10% CO_2_). Spectinomycin was added to BHI plates with a concentration of 1 mg/mL to select the *rnc*+ and *rnc*− strains as needed.

### Growth curve measurement

A single colony of each of the three strains was inoculated into the BHI medium and incubated in anaerobic conditions overnight (14–16 h). Then, the cultures were diluted to 1:20 with BHI medium and grown under anaerobic conditions for 2.5–3 h until the cells reached the mid-log phase (OD_600nm_ = 0.3–0.5) with constant turbidity in each group. The bacterial suspensions were transferred into sterile 96-well microtiter plates at a dilution of 1:100 and covered with sterile mineral oil in each well. Then, the growth of the strains was recorded using a monitoring system (BioTek, USA) for 24 h. Six biological replicates were used for each group in this study.

### Biofilm structure imaging and analysis

Scanning electron microscopy (SEM) was used to detect the structures of *E. faecalis* biofilms. The *E. faecalis* ATCC 29212 parent and mutant strains in their mid-log phases (OD_600nm_ = 0.3–0.5) were diluted to 1:100 with the BHI medium supplemented with 1% sucrose (BHIS). Bacterial suspensions were then transferred into a 12-well plate (2 mL per well), containing a round glass slide (14 mm in diameter). After 24 h of incubation, the biofilms were gently washed using phosphate buffered saline (PBS), and 2 mL of 2.5% glutaraldehyde was added to each well. The samples were then stored at 4 °C overnight. The biofilm samples of each group were serially dehydrated with 30%, 50%, 75%, 85%, 95%, 99% ethanol (v/v) for 15 min each time. There were three biological replicates for each group, which were examined at 1000×, 5000×, and 20,000× magnifications using SEM (Inspect Hillsboro, OR, USA).

Confocal laser scanning microscopy (CLSM) was performed to acquire fluorescence images and to determine the EPS/bacteria composition of *E. faecalis* biofilms. EPS was stained with Alexa Fluor® 647 (Invitrogen, Eugene, OR, USA), and bacteria cells were stained with Syto 9 Nucleic Acid Stain (Invitrogen, Eugene, OR, USA). CLSM (OLYMPUS, JAPAN) in order to observe the fluorescence images under a 20× objective lens. There were three biological replicates for each group, which were observed under three random observation fields. The three-dimensional biofilm images were reconstructed and the EPS/bacteria ratio was analyzed using Imaris 7.0.0 software. (Bitplane, Zurich, Switzerland).

### Crystal violet assay

Crystal violet assay was performed to quantitatively analyze the EPS matrix of biofilms. The biofilms of *E. faecalis* ATCC 29212 parent and mutant strains were incubated for 24 h at 37 °C under anaerobic conditions. After gently washing out the planktonic cells twice using PBS, 200 μL of 0.01% crystal violet (v/v) was added to each sample at room temperature for 10 min. After the careful removal of residual dye with running water, 33% acetic acid (v/v) was used to elute crystal violet, 37 °C, 150 rpm, 5 min. The OD_575_ values of the eluents were recorded. In order to evaluate the ability of drugs to remove *E. faecalis* biofilm EPS, 1 mL 5% NaOCl (v/v), 2%CHX (w/v), Pudilan (Pudilan keyanning antibacterial mouthwash, China), and PBS were added respectively to the biofilm samples and incubated for 10 min. Pudilan keyanning antibacterial mouthwash is a product mainly contains extracts of herbs in Pudilan formula. Therefore, we selected it to represent the Pudilan and detected its antibiofilm effect. Then, the drugs were gently washed using PBS. The procedures of crystal violet assays were the same as mentioned above.

### Detection of gene expression level

Total RNA was extracted from the mid-log phase bacteria using a MasterPure™ RNA purification Kit (Epicentre) following the manufacturer’s instructions. NanoDrop™ 2000c Spectrophotometer (Thermo Scientific, USA) was used to measure the concentration and purity of total extracted RNA. PrimeScript™ RT reagent Kit with gDNA Eraser (Perfect Real Time) (Takara, JAPAN) was used for the removal of genomic DNA and reverse transcription of RNA to cDNA. Quantitative real-time-PCR (RT-qPCR) was performed using LightCycler 480 (Roche, Switzerland). TB Green® Premix Ex Taq™ (Tli RNaseH Plus) (Takara, JAPAN) was used in the experiment according to the manual. The reaction process is as follows: 95 °C 30 s in the holding stage, then 95 °C 5 s and 60 °C 30 s for 40 cycles in the cycling stage, followed by melt curve stage and cool down. The reactions were carried out in triplicate. 16S rRNA was used as an internal standard and the relative expression level of the *rnc* gene was quantified using the 2^−ΔΔCT^ method. The RT-qPCR primer sequences are listed in Table 1.

**Table 1:** Real-time PCR primers

Primer	Nucleotide sequence
16SrRNA-F	5′-AAGCAACGCGAAGAACCTTA-3′
16SrRNA-R	5′-GTCTCGCTAGAGTGCCCAAC-3′
*rnc*−F	5′-TCCCAGAACTTCCAGAAGGA-3′
*rnc*−R	5′-GCGCCAACTTTTTGGTCTAA- 3′

### Antibacterial assays

The 24-h *E. faecalis* ATCC 29212 parent and mutant biofilm samples were prepared and the planktonic bacteria were removed using PBS. Each group of the biofilms were incubated with 1 mL 5% NaOCl, 2%CHX, Pudilan, and PBS respectively for 10 min. Then, the drugs were washed gently. PBS solution (1 mL) was added to each sample to form a uniform bacteria suspension. Then the bacteria suspension was diluted to different concentrations by PBS according to the antibiofilm ability of the drugs. The bacteria was diluted to 10^–2^ folds in the 5% NaOCl group, 10^–3^ folds in the 2%CHX and Pudilan groups and 10^–5^ folds in the PBS group. After mixing, 10 μL diluted bacterial suspension was dropped on BHI plate[24].

### Statistical analyses

Data analyses were performed using SPSS 26.0 (SPSS Inc., Chicago, IL, USA). One-way ANOVA method was used to identify the significance of variables’ effects. The Shapiro–Wilk test was applied and verified the data are normally distributed. Fisher's least significant difference was performed to compare the means of each group. Two-way ANOVA was applied to assess differences of the growth curves[25]. A *P* value < 0.05 was considered statistically significant.

## Results

### Down-regulation of the *rnc* gene inhibited bacterial growth and EPS synthesis

**Fig. 1:**
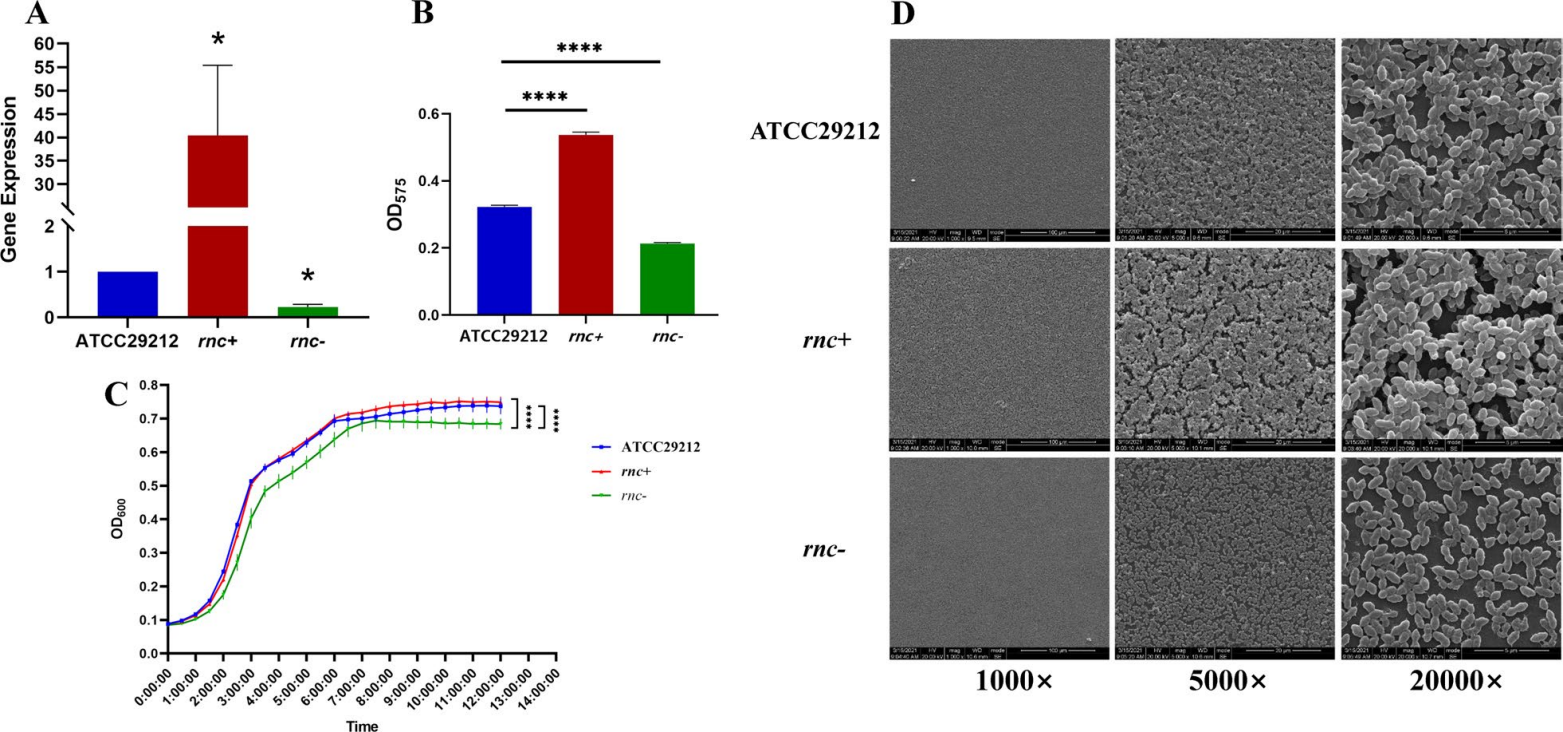
The *rnc* gene affects *E. faecalis* biological characteristics. **A** Quantitative RT-PCR analysis showed the *rnc* gene transcripts in *E. faecalis* ATCC29212, *rnc*+ and *rnc*−. The expression level of internal control 16S rRNA in ATCC29212 is set to 1.0. Experiments were performed in triplicate and are presented as the mean ± standard deviation. **B** Crystal violet assay showed the biomass of *E. faecalis* biofilms. **C** The growth curves of *E. faecalis* ATCC29212, *rnc* + and *rnc*−. **D** SEM images of 24-h cultured biofilms. (*P < 0.05, **P < 0.01, ***P < 0.001, ****P < 0.0001)

We tried different methods to reduce *rnc* expression level. Firstly, polymerase chain reaction ligation mutagenesis [26] was used to construct *rnc* deletion mutants without success. No colony growth on the antibiotics selective plate. The *rnc* gene seems to be essential for *E. faecalis* ATCC29212 viability. Then we introduced plasmids carrying *rnc* antisense sequences into *E. faecalis* ATCC29212. This method can effectively hinder the expression of *rnc* by pairing and forming a *rnc*− antisense *rnc* duplex structure. Similarly, the *rnc*+ mutant strain was made by introducing plasmids carrying *rnc* sequences [22]. The expression level of the *rnc* gene was identified using RT-qPCR (Fig. 1A). The results showed that as compared to the *E. faecalis* ATCC 29212 wildtype, the *rnc* expression level of the *rnc*+ strain increased by 40.13 times, while that of the *rnc*− decreased by 0.22 times. This confirmed the successful construction of *rnc*+ and *rnc*− mutant strains.

The growth curve of *rnc*+ and wildtype *E. faecalis* ATCC 29212 strains were similar; both reached the mid-log growth phase nearly the same time (Fig. 1C). However, the *rnc*− strain showed a slower growth rate under the same culture conditions, indicating its weaker proliferation capability. The *rnc*− strain spent a longer time reaching the mid-log growth phase and presented a lower OD_600_ value at the stationary phase. The average OD value of ATCC29212, *rnc*+ and *rnc*− were 0.737, 0.749 and 0.684 respectively. Statistical tests found significant difference between the *rnc*− strain and the other two species.

Crystal violet assays were performed to determine the differences in the total amount of EPS synthesis in the 24-h biofilms of wildtype, *rnc*+, and *rnc*− *E. faecalis* ATCC 29212 strains. As shown in Fig. 1B, the *rnc*+ strain showed significantly higher EPS productions as compared to the wildtype strain, while the *rnc*− strain showed significantly lower EPS contents (both *P* < 0.0001).

The morphology of the biofilms was evaluated using SEM (Fig. 1D). As compared to the wild-type strain, the biofilm of the *rnc*+ strain was rough and thick. Many deep gullies were observed under 5000× magnification. Then, under 20,000× magnification, the biofilm looked uneven and the bacterial cells aggregated through the extracellular matrix. On the contrary, the biofilm of *rnc*− strain contained a sparse matrix with fewer cracks on the surface. Under 20,000× magnification, the *rnc*− strain showed a loose combination between the matrix and bacterial cells.

**Fig. 2:**
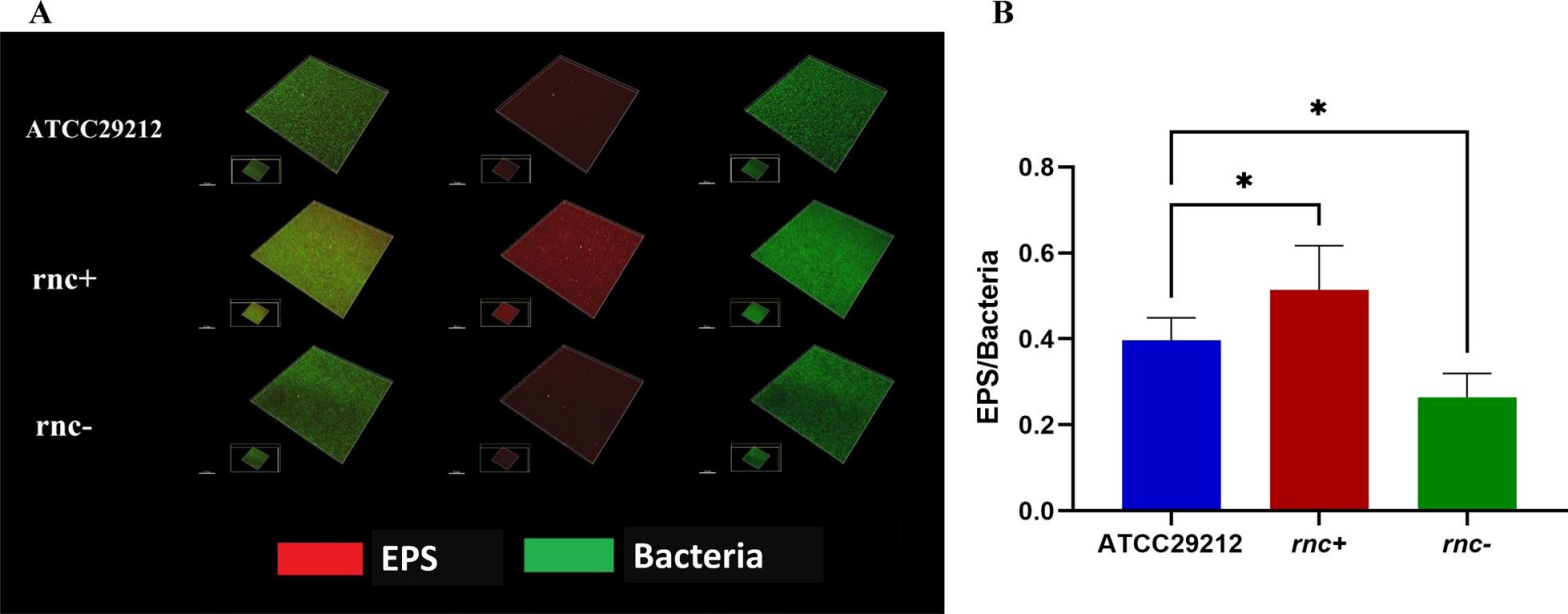
The *rnc* gene altered EPS and bacteria biomass ratio of biofilms. **A** The biofilm morphology was observed by CLSM. Double fluorescent labels marked bacteria (green, SYTO 9) and EPS (red, Alexa Fluor 647) respectively; scale bar = 50 μm. **B** The EPS/bacteria biomass ratio of *E. faecalis* ATCC29212, *rnc* + and *rnc − *. Three-dimensional reconstruction of the biofilms and quantitative data were performed by Imaris 7.0.0. (*P < 0.05)

The microscopic morphologies of the wildtype, *rnc*+, and *rnc*− strains were consistent with their performances under the naked eye. While preparing the samples, the *rnc*+ biofilms were found to be firmly attached to the glass slide and were more resistant to the water impact, while the *rnc*− biofilms were fragile. The CLSM showed that both the EPS and bacteria showed a thick accumulation in the *rnc*+ biofilm, while those in the *rnc*− biofilm showed decreased production and were scattered and unevenly distributed (Fig. 2A). Furthermore, the EPS/bacteria ratio in the *rnc*+ biofilm was higher than that of the wildtype strain (*P* < 0.05), while that of the *rnc*− strain was the lowest (*P* < 0.05) (Fig. 2B). Overall, the results consistently revealed that the *rnc* gene could positively regulate bacterial growth and biofilm formation in *E. faecalis*.

### Biofilms of *rnc* mutant strains showed an increased sensitivity to disinfectants

**Fig. 3:**
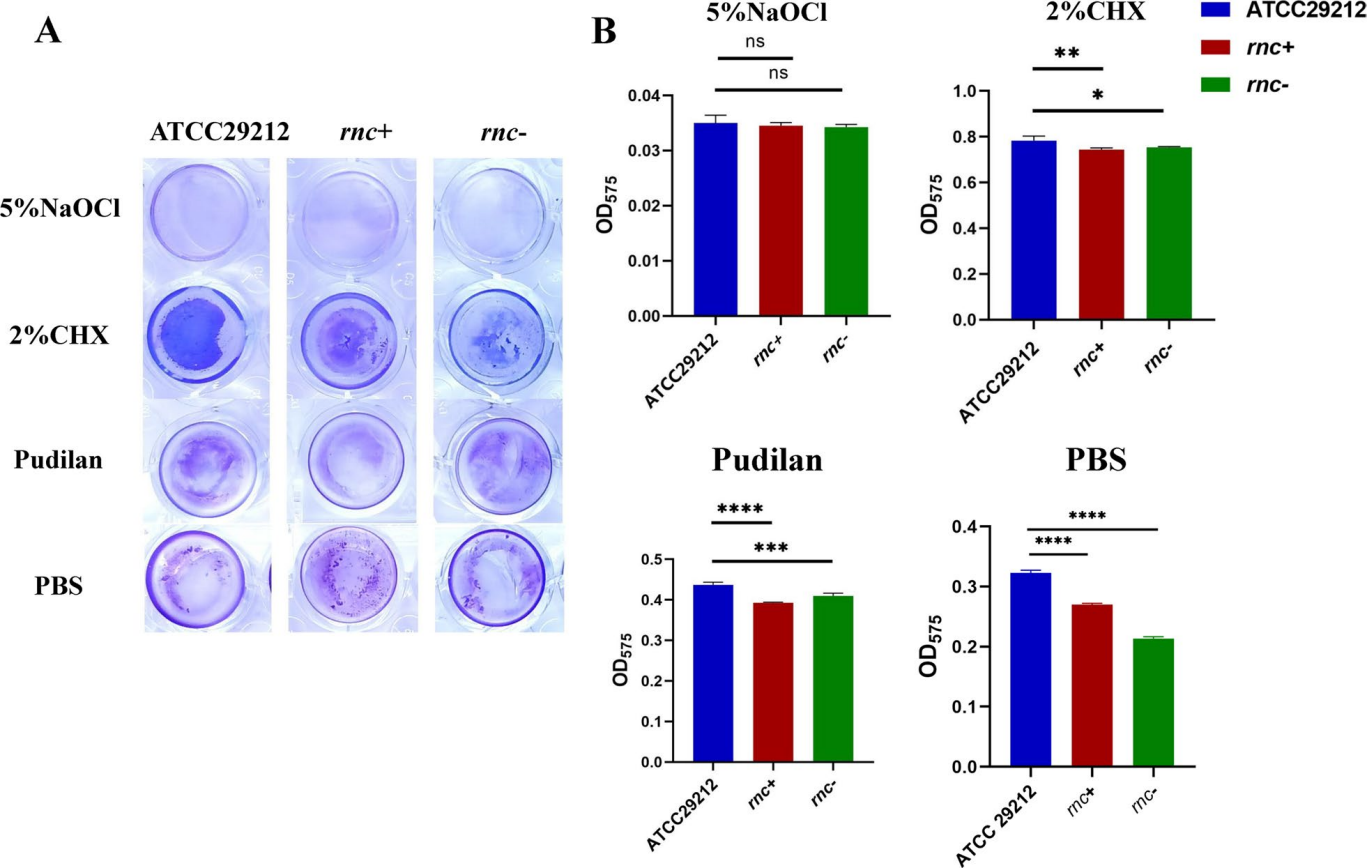
*E. faecalis* biofilm resistance towards several antibacterial agents was regulated by the *rnc* gene. **A** Crystal violet assay showed the residual biomass of *E. faecalis* biofilms after treated by antibacterial agents. **B** Quantitative data showed the difference in the amount of residual biofilm of *E. faecalis* ATCC29212, *rnc*+ and *rnc*−. (ns = no significance, *P < 0.05, **P < 0.01, ***P < 0.001, ****P < 0.0001)

**Fig. 4:**
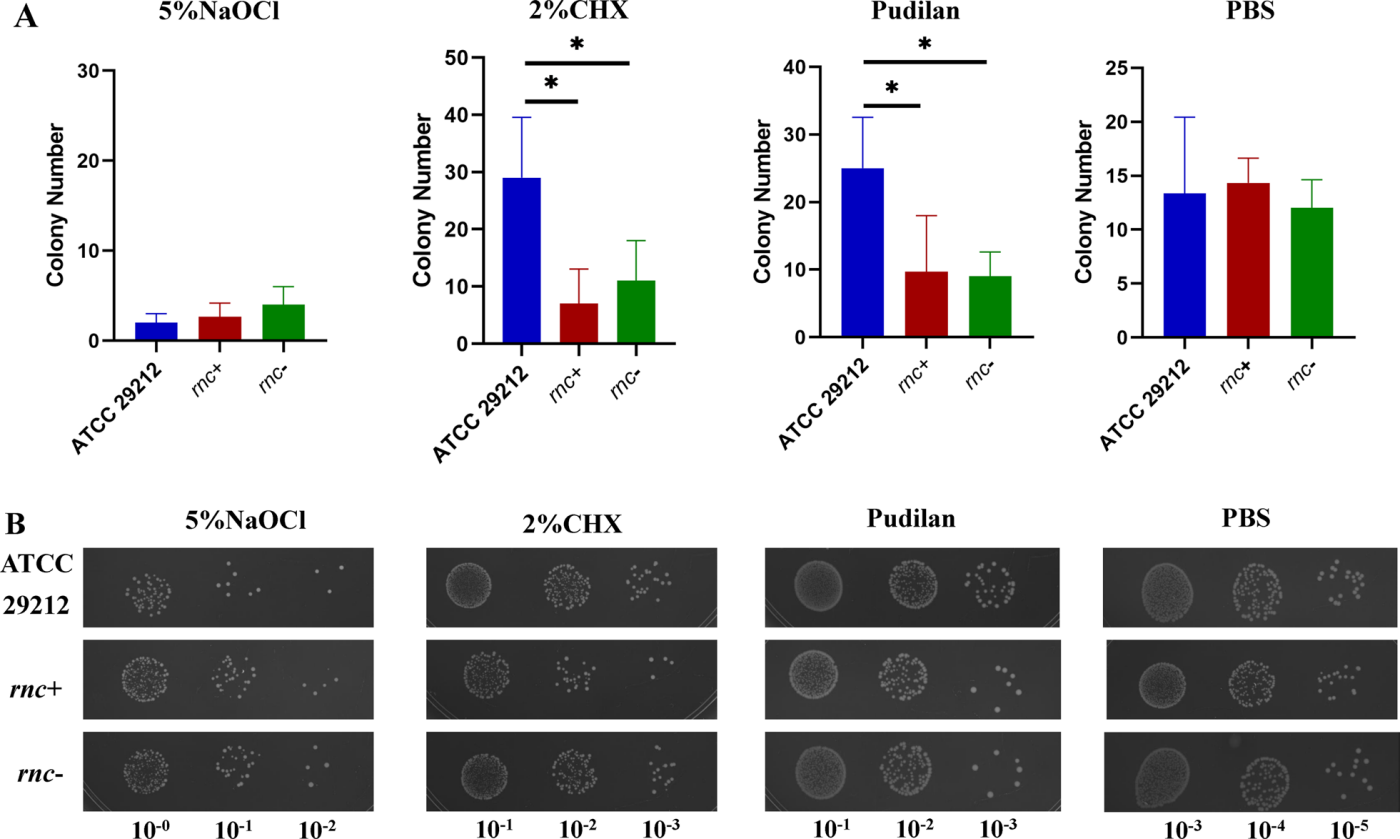
the *rnc* gene altered the viable count in *E. faecalis* biofilms. **A** The colony number of *E. faecalis* biofilms after treated by antibacterial agents. (*P < 0.05). **B** The appearance of the colony from *E. faecalis* biofilms after treated by antibacterial agents. The dilution modulus was marked below the pictures

In order to compare the sensitivities of *E. faecalis* ATCC 29212 wildtype and *rnc* mutant strains to the different antibacterial agents, crystal violet assays were performed to quantify the EPS residues in the biofilms after treatment with the respective antibacterial agents. 5% NaOCl was set as a positive control. After incubating for 10 min with 5% NaOCl, all the three biofilms were almost eliminated with no significant differences (Fig. 3A). Interestingly, the *rnc*+ and *rnc*− groups showed lower EPS residues as compared to the wildtype strains after treatment with 2% CHX, Pudilan, and PBS, suggesting that the biofilms of *rnc* mutant strains were more sensitive to these antibacterial agents (Fig. 3B). Particularly, Pudilan showed better anti-biofilm activity as compared to the 2% CHX. Furthermore, the number of active bacteria in the wildtype and *rnc* mutant biofilms were compared after treatments with different drugs (Fig. 4). Due to the different antibiofilm ability of the agents, we diluted the bacteria suspension to 10^–2^ folds in 5% NaOCl group, 10^–3^ folds in 2% CHX and Pudilan group, 10^–5^ folds in PBS group. As a positive control, 5% NaOCl showed the strongest antibiofilm ability towards the three strains with no significant difference among the ATCC29212, *rnc*+ and *rnc*− groups. In the 2% CHX and Pudilan group, there are significantly more colonies of ATCC29212 than *rnc*+ and *rnc*−. The PBS treated groups showed similar column number.

## Discussion

The pathogenic biofilms of *E. faecalis* are closely associated with periapical periodontitis. The removal of *E. faecalis* biofilms is crucial for avoiding the failure of root canal treatments. Due to the complexity of the root canal system [27], chemical irrigation agents are supposed to disinfect the root canal, especially where the mechanical preparations cannot reach it. The characteristics of the root canal irrigation agents determine their effects. As irrigation agents, 5% NaOCl and 2% CHX are effective and widely used. However, due to the irritation to the periapical tissue [28], 5% NaOCl should be applied with caution in the clinic. CHX has also shown some side effects, such as irritation to the oral mucosa, causing a burning sensation, and alteration of taste perception [29]. Therefore, the development of alternative drugs and improvement of bacterial susceptibility has been continuously sought. Pudilan is a commercial TCM made up of herbal extract. Pudilan keyanning mouthwash products contain Pudilan extract and 0.03%–0.06% cetylpyridinium chloride (CPC). The CPC (0.03–0.06%) has been reported with little antibacterial effects, which were weaker than 0.12% CHX [15]. In the current study, Pudilan exhibited a stronger EPS removing effect as compared to 2% CHX and was more effective on the *rnc*+ and *rnc*− strains. In the colony number assay, Pudilan and 2%CHX also showed stronger antibacterial effect on *rnc*+ and *rnc*− strains than ATCC29212. Overall, Pudilan preliminary showed an excellent anti-biofilm effect on *E. faecalis* but still needs further investigations.

Similar to *E. faecalis*, another Gram-positive coccus bacteria *S. mutans*, relies on the formation of stable biofilm to acquire resistance and cause virulence in the mouth. Preliminary studies have shown that the *rnc* deletion mutation could repress *S. mutans* cariogenicity in rat models, and the weakness of biofilm was attributed to the reduced EPS production and bacterial adhesion [10]. As *rnc* is a highly conserved gene, we proposed *rnc* as a target to eliminate the *E. faecalis* biofilms. The results demonstrated its excellent regulatory effects on biofilm metabolism and drug sensitivity. The *rnc* overexpression strain *rnc*+ and *rnc* low-expression strain *rnc*− were established and the effects of its *rnc* expression level on the growth status of *E. faecalis* were observed. The *rnc*+ strain showed a normal growth rate and formed a thriving biofilm, while the *rnc*− strain showed a delayed growth rate and fragile biofilm. These results revealed that the *rnc* gene could positively regulate the bacterial growth and formation of biofilm in *E. faecalis*, which was consistent with our hypothesis. The *rnc* gene encodes RNase III, which regulates gene expression at the post-transcription level [30]. Therefore, the *rnc* expression level might have a profound impact on the phenotypes of bacteria and biofilms. On the other hand, antisense *walR* as a post-transcriptional modulator, has been proven to regulate bacterial growth, virulence and EPS synthesis and aggregation. This is a successful precedent for post-transcriptional level regulation as an anti-biofilm target in *E. faecalis* [31].

In order to evaluate the ability of the three strains to drug resistance, the EPS residues and their colony number in biofilms after incubation were tested with different drugs. As expected, the 5% NaOCl group showed the least OD_575_ absorbance as expected, followed by PBS, Pudilan, and 2% CHX group. The increased OD in the Pudilan and 2% CHX groups as compared to the PBS group might be due to the increased light absorbance by the biofilm pigmentation caused by its color. After treatment, the EPS residues in the biofilms of *rnc* mutant strains were less than those of the wild-type strain. It was concluded that the *rnc*− strain showed weakened drug resistance due to its thin and barren extracellular matrix. Interestingly, the thick *rnc*+ biofilm also showed increased sensitivity to the antibacterial drugs. The SEM and CLSM observation of the thick and uneven *rnc*+ biofilm suggested that this unevenness of the biofilm allowed the antibacterial drugs to penetrate, thereby showing their antibacterial effects. The *rnc* interference strategy not only reduced the EPS metabolism of *E. faecalis* biofilms but also made the biofilms more fragile, resulting in increased drug susceptibility. In order to comprehensively understand the regulatory effects of *rnc* on biofilm formation and their mechanisms, further studies of related genes, including *epa*I/*epa*OX [32], *gelE,* and *esp* [33] are required. Approaches to *rnc* gene regulation rather than the antibiotic use and development of resistance might be more in line with ecological regulation.

However, there are some limitations in this study. First, the biofilm models used in the experiment may not fully reflect the state in the disease. This was an in-vitro experiment and the biofilm samples were 24-h early mature biofilms. Ali et al*.* showed that the substrate-conditioning substances and biofilm age could affect the components of the cellular and extracellular matrix of *E. faecalis* biofilms [34]. Moreover, we failed to delete the *rnc* gene from genomic DNA either through chemical transformation or electroporation method, but the *rnc* deletion mutant was constructed in *E. faecalis* V19 [35], which is a plasmid-cured derivative of the vancomycin-resistant clinical isolate V583[36]. The characteristic differences between type strain ATCC29212 and drug-resistant clinical strain V19 may explain the failure to knock out the *rnc* gene in ATCC29212. Moreover, the biofilm phenotype and drug resistance changes of the *rnc*− strain were obvious enough to judge the trend of the results. Therefore, we take the *rnc*− strain to observe the regulation effect of the *rnc* gene. The *rnc*− strain was constructed by transforming a shuttle plasmid loaded with an *rnc* antisense RNA sequence. Here are other possible hypotheses for failure to construct *rnc* deletion mutant strain. (1) The exogenous plasmids are abnormally expressed in bacteria; therefore, the mutant strains cannot survive on a selective medium. (2) The thick capsule of membrane shuts long-chain DNA out. (3) The transformation methods need further optimization. Although the *rnc−* strain showed decreased growth, the copy number variation might cause genetic and expression instability [37]. In brief, more advanced biofilm models and mutant strains are expected to be used in exploring anti *E. faecalis* targets.

## Conclusions

In this study, the *rnc* overexpression and low-expression mutant strains of *E. faecalis* were successfully constructed. The biological features of *rnc* mutant strains and their sensitivity towards typical root canal irrigation agents and TCM fluid Pudilan were evaluated. This study revealed that the overexpression of *rnc* could promote bacterial growth and EPS synthesis, and vice versa. However, the altered *rnc* expression level could break the balance, forming a vulnerable biofilm. The altered biofilm structure made it more sensitive to the antibacterial agents, allowing for a decrease in antibiotic use and resistance. Taken together, these data suggested the *rnc* gene as a biofilm regulatory target and provided evidence for the antibacterial potential of Pudilan, providing a novel strategy for the management of root canal system and apical infection.

## Supplementary Information

**Additional file 1**. The *rnc* gene coding sequence, the reverse complementary sequence and promotor sequence are provided in supplemental material.

## Data Availability

The datasets used and/or analysed during the current study are available from the corresponding author on reasonable request.

## Abbreviations

*E. faecalis*: *Enterococcus faecalis*
EPS: Extracellular polysaccharide
*rnc*+: *rnc* Gene overexpression strain
*rnc*−: *rnc* Gene low-expression strain
SEM: Scanning electron microscopy
CLSM: Confocal laser scanning microscopy
NaOCl: Sodium hypochlorite
CHX: Chlorhexidine
RNase III: Ribonuclease III
msRNAs: MicroRNA-size small RNAs
TCM: Traditional Chinese medicine
CSP: Competence-stimulating peptides
BHI: Brain heart infusion
PBS: Phosphate buffered saline
RT-qPCR: Quantitative real-time-PCR
CPC: Cetylpyridinium chloride
