# Supplementary material for: Enterococcus faecalis rnc gene modulates its susceptibility to disinfection agents: a novel approach against biofilm
Source: BMC Oral Health. 2022 Sep 20;22:416. doi: 10.1186/s12903-022-02462-1 (PMC9490916; doi:10.1186/s12903-022-02462-1)
Supplement: Supplementary file 1 — Additional file 1. The rnc gene coding sequence, the reverse complementary sequence and promotor sequence are provided in supplemental material. [file 12903_2022_2462_MOESM1_ESM.docx]

***Enterococcus faecalis* *rnc* gene modulates its susceptibility to disinfection agents****: a novel approach against biofilm**

Mengying Xia, Niya Zhuo, Shiru Ren, Hongyu Zhang, Yingming Yang, Lei Lei, Tao Hu.

The promotor sequence of *rnc* and antisense *rnc* on pDL278 plasmid.

ACAACGTTCCGCTGATTTAATAGAGATAGAACGAGGTCGGGACAAAAGTGTTTAACTCCGAGAAATAAGGAGGAGTTGCTTCTGTTCCCGCCGTTTATCAGTTTTTGAGCGTGGCGCAAAAATCCAAAGTGGTTTTTGTCCCACGCTCTTTCTATCTCTTACGTAAACAAACGCTTTCATCTCAGTCATAGCTCTATTTATTTATTTTTTTCTAAAAAATAATGAGAACACAGTGAATAACAGGCTTTTTCTGAATAGAACCATTTAAAAAAAGTGAAAAAAAAAGTATAATGAAACAAGGTGTAAATAAATGTAAAGGAATGTGGAGA

The coding sequence of *rnc* gene is as follows.

(Resourse: https://www.ncbi.nlm.nih.gov/gene/?term=rnc+Enterococcus+faecalis)

ATGGACAATCAGTTAACAACAGAGTTAAAAGAACGTTACGGCATTGTTTTCCATGATGTCAATCTATTAGAGCAAGCTTTTACTCATTCATCCTATGTGAATGAGCATCGCTATTTAAAATTATCCGATAATGAACGTCTTGAATTTTTAGGAGATGCAGTTTTAGAATTAATTGTTTCACAATATTTGTATTTAAAATTCCCAGAACTTCCAGAAGGAAAATTAACGAAGATGCGCGCAGCCATCGTTCGGGAAGATAGTTTAGCCAAATTTGCGAAAGAATGTCACTTCGACAACTACATTTTATTAGGTAAAGGAGAAGAAGCATCGGGCGGACGAACACGTGCATCATTATTATGTGACTTATTTGAAGCCTTTTTAGGTGCCCTCTACTTAGACCAAAAAGTTGGCGCAGCCAAGAAATTTATTGAAGACGTTATTTTTCCGAAAATTGATGCCGGTGCTTTTTCACATGAGATGGATCACAAAACACAATTACAAGAAGTTTTACAACGCAAAGGCGATGTTTCAATTGAATATCGCTTAATTAAAGAAGAAGGCCCTGCTCATGACCGCACCTTTTTCACTGAAGTTTACATGAATGGTGAACTCATTGGGTTAGGCCAAGGAAAATCGAAGAAGTTAGCTGAACAGGATGCCGCTGAGCGGGCACTGAAAAGTATTCCTCAGTAA

The antisense *rnc* sequence is as follows.

(Designed by: <http://www.bioinformatics.org/sms/rev_comp.html>)

TTACTGAGGAATACTTTTCAGTGCCCGCTCAGCGGCATCCTGTTCAGCTAACTTCTTCGATTTTCCTTGGCCTAACCCAATGAGTTCACCATTCATGTAAACTTCAGTGAAAAAGGTGCGGTCATGAGCAGGGCCTTCTTCTTTAATTAAGCGATATTCAATTGAAACATCGCCTTTGCGTTGTAAAACTTCTTGTAATTGTGTTTTGTGATCCATCTCATGTGAAAAAGCACCGGCATCAATTTTCGGAAAAATAACGTCTTCAATAAATTTCTTGGCTGCGCCAACTTTTTGGTCTAAGTAGAGGGCACCTAAAAAGGCTTCAAATAAGTCACATAATAATGATGCACGTGTTCGTCCGCCCGATGCTTCTTCTCCTTTACCTAATAAAATGTAGTTGTCGAAGTGACATTCTTTCGCAAATTTGGCTAAACTATCTTCCCGAACGATGGCTGCGCGCATCTTCGTTAATTTTCCTTCTGGAAGTTCTGGGAATTTTAAATACAAATATTGTGAAACAATTAATTCTAAAACTGCATCTCCTAAAAATTCAAGACGTTCATTATCGGATAATTTTAAATAGCGATGCTCATTCACATAGGATGAATGAGTAAAAGCTTGCTCTAATAGATTGACATCATGGAAAACAATGCCGTAACGTTCTTTTAACTCTGTTGTTAACTGATTGTCCAT

The sequence of *rnc*, antisense *rnc* and promotor region were synthesized by Sangon Biotech (Shanghai, China).
